# Supplementary material for: Potential Role of Soluble Toll-like Receptors 2 and 4 as Therapeutic Agents in Stroke and Brain Hemorrhage
Source: Int J Mol Sci. 2021 Sep 15;22(18):9977. doi: 10.3390/ijms22189977 (PMC8470802; doi:10.3390/ijms22189977)
Supplement: Supplementary file 1 [file ijms-22-09977-s001.zip › ijms-1344178-supplementary.pdf]

Table S1. Preclinical Table

Reference	Disease Studied & Model	Avg. Age/Age range/Weight range, Species Tested, Population Size, Sex Predominance, Duration	TLRs Studied	Drug/Treatments	Anatomical	Functional
Figueiredo et al., Characterization of heme as activator of Toll-like receptor 4, (1)	eg. SCD & ICH hemolytic disease: Incubated with heme (30 μM for 1h)	n/d<br>C57Bl/6 (WT), Tlr2$^{-/-}$, Cd14$^{-/-}$, Myd88$^{-/-}$, and C3H/HeJ (TLR4$^{d/d}$) mouse macrophages<br>n/d<br>n/d<br>18h	TLR4	In vitro: heme (3–30μM), protoporphyrin IX (PPIX, 3–30μM), LPS (100ng/mL), polymyxin B (1μg/mL)	Heme induced TNF-α expression in macrophages compared to control, although less than LPS. LPS-induced expression of TNF-α was reduced by polymyxin B, but not heme-induced expression. Heme-induced expression of TNF-α was reduced by PPIX and other mesoporphyrin analogues, but LPS-induced expression was not. Heme-induced expression of TNF-α was not found in Cd14$^{-/-}$, Myd88$^{-/-}$, or TLR4$^{d/d}$ macrophages, but it was found in Tlr2$^{-/-}$ macrophages.	n/d
Hanafy, The role of microglia and the TLR4 pathway in neuronal apoptosis and	SAH, in vitro: incubated with hemin; in vivo: injected with 60μl heterologous	10–12wk<br>C57BL/6 (WT), TLR4$^{-/-}$, TRIF$^{-/-}$, and MyD88$^{-/-}$ mice<br>n=6 (WT), 6 (TLR4$^{-/-}$), 6 (TRIF$^{-/-}$), and 6 (MyD88$^{-/-}$)	TLR4	Autologous blood (60μl)<br>In vitro: hemin (40μM)	WT group had an increase in vasospasm compared to sham on postoperative day (POD) 5. Microglia were the largest source of TLR4 expression after injury.	n/d

vasospasm after subarachnoid hemorrhage, (2)	blood to simulate SAH	100% M<br>15d			LPS was found to create neural injury but not as much as the SAH model.<br>TLR4$^{-/-}$ group had decreased markers of SAH, such as neuronal apoptosis, compared to WT.<br>TRIF$^{-/-}$ had increased neuronal apoptosis ($p<0.03$) and vasospasm ($p<0.01$) on POD 7 compared to TLR4 KO.<br>MyD88$^{-/-}$ group had increased neuronal apoptosis on POD 15 ($p<0.04$) compared to TLR4 KO.	
Liu et al., Deficiency of Tenascin-C Alleviates Neuronal Apoptosis and Neuroinflammation After Experimental Subarachnoid Hemorrhage in Mice, (3)	SAH, puncture at the bifurcation of the left anterior and middle cerebral arteries	10–12wk<br>25–30g<br>C57BL/6 (WT) and TN$^{-/-}$ mice<br>n=25 (WT) and 25 (TN$^{-/-}$)<br>100% M<br>24h	TLR4	n/d	SAH significantly increased expression of TLR4 and related IL pathway in the ipsilateral hemisphere compared with both sham groups ($p<0.05$).<br>SAH+TN$^{-/-}$ group had attenuated TLR4 expression compared to SAH+WT group ($p<0.05$).	Beam balance test and neurological scores were significantly impaired in the SAH+WT groups compared with sham groups 24h postoperative ($p<0.05$)<br>SAH+TN$^{-/-}$ group had improved balance beam test and neurological

						score compared to SAH+WT ($p<0.05$)
Wang et al., Toll-like receptor 2/4 heterodimer mediates inflammatory injury in intracerebral hemorrhage, (4)	ICH, autologous blood (30µl), the same volume of Hb (20µM), or bacterial collagenase (0.075 U in 1µl) injected in vivo	n/d<br>20–23g (WT), 21–22g (KO)<br>C57BL/6 (WT), TLR2$^{-/-}$, TLR1$^{-/-}$, MyD88$^{-/-}$, TRIF$^{-/-}$, and TLR6$^{-/-}$ mice<br>n/d<br>100% M<br>5d	TLR2/TLR4, TLR6	Saline (1µl, vehicle)	Hb induced the most inflammatory activity when TLR2 and TLR4 were both present. TLR2/TLR4 heterodimer formation was detected in WT and TRIF$^{-/-}$ mice but not in MyD88$^{-/-}$ mice.	Decreased NDS in TLR2$^{-/-}$ mice ($p<0.01$) compared to WT. TLR2$^{-/-}$/TLR4$^{-/-}$ mice had a lower NDS than TLR2$^{-/-}$ or TLR4$^{-/-}$ mice alone.
Wang et al., TLR7 (Toll-Like Receptor 7) Facilitates Heme Scavenging Through the BTK (Bruton Tyrosine Kinase)-CRT (Calreticulin)-LRP1 (Low-Density Lipoprotein Receptor-Related Protein-1)-Hx (Hemopexin) Pathway in Murine Intracerebral	ICH, bacterial collagenase (0.075 U dissolved in 0.5µl saline) injection	8wk<br>30±5g<br>CD1 mice<br>n=177 (Experiment 1), 54 (Experiment 2)<br>100% M<br>28d	TLR7	Experiment 1: Sham, ICH + vehicle (0.5µl saline), ICH + imiquimod (TLR7 agonist)<br>Experiment 2: Same as experiment 1, plus the addition of an ICH+ODN2088 (TLR7 antagonist) group, ICH+LFM-A13 (BTK inhibitor), ICH+thapsigargin (CRT agonist), ICH+ODN2088+thapsigargin groups	Experiment 1: Although higher doses of imiquimod resulted in smaller hematoma than ICH+vehicle ($p<0.05$), only low dose 2.5 ug/g treatment resulted in less BWC and BBB permeability ($p<0.05$). Less iron content in the ipsilateral brain hemisphere on day 28 post-ICH in the imiquimod treated group ($p<0.05$).<br>Experiment 2: TLR7, BTK, CRT, LRP1, and Hx were elevated in ICH+vehicle compared to sham ($p<0.05$) and increased to a greater degree in	Low-dose imiquimod had higher scores than the ICH+vehicle group in all neurological tests ($p<0.05$). Functional outcome: Low-dose imiquimod group continued to have increased neurological scores compared to the ICH+vehicle group at 23–28 days ($p<0.05$).

Hemorrhage, (5)					ICH+imiquimod groups than ICH+vehicle ($p<0.05$). ODN2088 group had decreased expressions of TLR7, BTK, CRT, LRP1, and Hx compared with ICH+vehicle ($p<0.05$). LFM-A13 group resulted in reduced BTK, CRT, LRP1, and Hx expression compared with ICH+vehicle ($p<0.05$). ICH+thapsigargin resulted in increased expression of CRT, LRP1, and Hx. ICH+ODN2088+thapsigargin reduced TLR7, BTK, LRP1, and Hx expression but not CRT expression compared to ICH+vehicle ($p<0.05$).	
Zhang & Zhang, Rnf112 deletion protects the brain against intracerebral hemorrhage (ICH) in mice by inhibiting TLR-4/NFκB pathway, (6)	ICH in vivo: ICH (25-μL autologous blood injection) In vitro: OxyHb (10μM)	8–10wk 20–22g C57BL/6J (WT), Rnf112$^{-/-}$, and TG (Transgenic) mice infected with Rnf112 overexpression adenovirus n/d 100% M 3d	TLR4	In vivo: sham (0.9% saline), ICH in vitro: LPS (TLR4 agonist, 10μM), OxyHb (10μM)	In vivo: Rnf112 expression significantly decreased in the ICH+WT group compared to sham, reaching the lowest peak at 24h ($p<0.001$). In vitro: treatment of astrocyte and microglial cells also show decreased Rnf112 expression, reaching a peak at 24h for astrocytes and 12h for microglia ($p<0.001$ and $p<0.001$,	WT+ICH group showed a greater NDS than the sham group ($p<0.01$). Rnf112$^{-/-}$+ICH group showed a greater NDS than WT+ICH group ($p<0.01$). Overexpression of Rnf112 in

					respectively). $Rnf112^{-/-}$+ICH groups had increased BWC compared to the WT+ICH group (p<0.05). $Rnf112^{-/-}$+ICH group showed increased expression of TLR4 (p<0.05) and MyD88 (p<0.01) within hematoma compared to WT+ICH group. In vivo: TG+ICH group had decreased inflammatory marker expression (p<0.01) and BWC (p<0.05) than the negative control. In vitro: Overexpression of Rnf112 resulted in decreased expression of TLR4/MyD88 pathway.	TG mice resulted in a lower NDS than the negative control (p<0.01).
Zhou et al., Fisetin alleviates early brain injury following experimental subarachnoid hemorrhage in rats, possibly by suppressing TLR 4/NFκB signaling pathway, (7)	SAH, 300μl autologous blood from the femoral artery to prechiasmatic cistern	n/d<br>280–320g<br>Sprague–Dawley rats<br>n=211<br>100% M<br>72h	TLR4	sham, sham+high dose (50mg/kg) fisetin, SAH, SAH+vehicle, SAH+low dose (25mg/kg) fisetin, SAH+high dose fisetin	BWC decreased in SAH+high dose fisetin group compared to SAH group at 24h and 72h (p<0.01 and p<0.05, respectively). SAH+high dose fisetin group showed decreased TLR4 expression compared to SAH group (p<0.01). SAH+high dose fisetin group showed decreased expression of proinflammatory cytokines compared to	SAH+high dose fisetin decreased neurological score at 24h and 72h compared to SAH group (p<0.01 and p<0.05, respectively). No significant difference in mortality rate.

					SAH group. SAH+high Dose fisetin group showed increased expression of ZO-1 (a protein needed to maintain BBB impermeability) compared to SAH group ($p<0.05$).	
Zhang et al., Astaxanthin mitigates subarachnoid hemorrhage injury primarily by increasing sirtuin 1 and inhibiting the Toll-like receptor 4 signaling pathway, (8)	SAH, 0.35mL autologous blood	8–10wks (mice) 250–300g (rats) Sprague-Dawley rats, C57BL/6 (WT) mice, and TLR4-/- mice 100% M 3d	TLR4	In vitro: oxyHb (10μM) + ATX (5, 10, and 50 μM), In vivo: sham, sham+ATX, SAH, SAH+0.01mM ATX, SAH+0.1mM ATX, and SAH+0.2mM ATX. 0.1 ATX administered at 30min, 4h, or 8h.	0.1 and 0.2mM ATX treatment significantly decreased inflammatory cytokine expression at 6h and 24h post-SAH compared to SAH+vehicle group ($p<0.05$ for each). 0.1 and 0.2mM ATX treatment significantly increased neuron cell count at 72h post-SAH compared to SAH+vehicle ($p<0.05$ for both). SAH+ATX (0.1mM) group showed decreased expression of HMGB1, TLR4, and MyD88 compared to SAH+vehicle group 24h post-SAH ($p<0.05$). SAH+ATX(0.1mM) group showed increased expression of SIRT1 compared to SAH+vehicle ($p<0.05$). SAH+ATX+Sirtinol (SIRT1 inhibitor) showed reversal of changes in	0.1 and 0.2mM ATX treatment significantly increased neurological score and rotarod performance at 24h and 48h and significantly reduced body weight loss at 72h compared to SAH+vehicle group ($p<0.05$ for each). SAH+ATX+KO mice group did not have significantly different neurological impairment compared to SAH+vehicle+ KO mice.

					protein expression (i.e., no s.d. in any protein expression). SAH+vehicle+KO mice group had decreased neuronal apoptosis compared to SAH+vehicle mice (p<0.05); however, SAH+ATX+KO mice still demonstrated less apoptosis than the SAH+vehicle+KO mice group (p<0.05). SAH+ATX group had significantly decreased apoptotic markers (p<0.05) and BWC (p<0.05) compared to SAH+vehicle. Previously mentioned anatomical outcomes were present in 30min and 4h post-SAH treatment, but not 8h post-SAH treatment. In vitro, ATX treatment showed less expression of TLR4 and inflammatory cytokines in cells treated with oxyHb.	Improvements in neurological function were present in treatments at 30min and 4h post-SAH but not 8h post-SAH.
Huang et al., Heme oxygenase-1 protects rat liver against warm	Liver ischemia/reperfusion, traumatic clip of hepatic artery and portal vein for 75min	n/d<br>220–250g<br>Sprague–Dawley rats<br>n=16<br>100% M<br>6h postsurgery	TLR2/TLR4	sham (0.5ml saline), Liver I/R (surgery+0.5ml saline) group, I/R+CoPP (HO1 inducer) group,	I/R+CoPP group showed decreased (p<0.05) enzymatic markers of liver injury, whereas I/R+ZnPP showed increased (p<0.05)	n/d

ischemia/reperfusion injury via TLR2/TLR4-triggered signaling pathways, (9)				I/R+ZnPP (HO inhibitor) group	enzymatic markers compared to I/R group. I/R+CoPP group had decreased ($p<0.05$) Suzuki scores, whereas I/R+ZnPP had greater ($p<0.05$) scores than I/R group. HO1, TLR2, and TLR4 increased in I/R+ZnPP group compared to I/R+CoPP group based on Western blot. I/R+CoPP had increased expression of TLR regulators compared to I/R ($p<0.05$ for each). I/R+ZnPP had decreased expression for all TLR regulators except SOCS-1 compared to I/R ($p<0.05$).	
Wu et al., Peroxisome proliferator-activated receptor gamma agonist rosiglitazone attenuates oxyhemoglobin-induced Toll-like receptor 4 expression in vascular smooth muscle cells, (10))	SAH, 10μM oxyhemoglobin treatment	3–4wk Sprague–Dawley rats (vascular smooth muscle cell culture)<br>n/d<br>100% M<br>48h	TLR4	Sham (DMSO vehicle only), OxyHb (10μM+DMSO), OxyHb+rosiglitazone (PPARγ agonist, 10μM), OxyHb+rosiglitazone+Gw9662 (PPARγ antagonist, 10μM)	OxyHb+rosiglitazone group showed decreased TLR4 expression compared to OxyHb group ($p<0.01$). Difference disappeared in OxyHb+rosiglitazone+GW9662 group. Similar results were found in immunohistochemical staining and Western blot. OxyHb+rosiglitazone group showed decreased TNF-α expression	n/d (in vitro only)

					compared to OxyHb group ($p<0.01$). Difference disappeared in OxyHb+rosiglitazone+GW9662 group.	
García-Culebras et al., Role of TLR4 (Toll-Like Receptor 4) in N1/N2 Neutrophil Programming After Stroke, (11)	Ischemic stroke, pMCAO	8–10wk B6.C57BL/6J (WT) mice and B6.B10ScN–TLR4lps–del/JthJ ($TLR4^{-/-}$) mice n=6–9 (each group) 100% M 48h post–pMCAO	TLR4	pMCAO+heterogeneous bone marrow replacement	$TLR4^{-/-}$ mice had smaller brain infarct than WT 24h post-MCAO ($p<0.05$) and 48h post-MCAO ($p<0.05$). $TLR4^{-/-}$ had higher neutrophil infiltration in the brain ($p<0.05$), whereas WT had more neutrophil presence in the blood ($p<0.05$). Moderate linear correlation between neutrophil infiltration and infarct volume; positive for WT ($p=0.0121$), negative for $TLR4^{-/-}$ ($p=0.0466$). In the chimeric host, non-TLR4 expressing neutrophils were more likely to infiltrate the brain after stroke ($p<0.05$). Results were similar in a trial of chimeric mice, which only express TLR4 in microglia and not in the myeloid lineage. Lack of TLR4 in myeloid lineage also causes neutrophils to express a greater	Not measured

					percentage of N2 than control ($p<0.05$).	
Vinchi et al., Hemopexin therapy reverts heme-induced proinflammatory phenotypic switching of macrophages in a mouse model of sickle cell disease, (12)	Hemolytic disease (eg. SCD & ICH), 70µmol/kg hemin injected intravenously	2–3mth<br>B6J or SV129 (WT) mice, TLR4 KO mice, Hx KO mice, HbS Knock–in Mice<br>n/d<br>n/d<br>In vivo: 3wks, In vitro: 15h	TLR4	In vitro: 5 to 15µM heme or Zinc-mesoporphyrin bound with 5 to 15µM albumin (human or murine) or Hx. In vivo: hemin 70µmol/kg	In vivo: $Hx^{-/-}$+Heme group had higher levels of HO1 ($p<0.05$), L-Ferritin ($p<0.01$), FPN ($p<0.01$), and IL-6 ($p<0.05$) than WT+heme group, suggesting less heme breakdown. Heme causes macrophages to express M1 phenotype, regardless of previous cell differentiation. Hx increased MMP-9 expression in the liver. Similar results in HbS (sickle cell) mice.<br>In vitro: Hx+heme had lower levels of HO1 ($p<0.001$), ferritin ($p<0.001$), and FPN ($p<0.01$) at 10h incubation and lower levels of ROS ($p<0.001$), TNF-α ($p<0.01$), and IL-6 ($p<0.01$) at 15h than Albumin+heme. Heme+TAK-242 (TLR4 inhibitor) and Heme+NAC (antioxidant) had attenuated heme-induced M1 polarization of macrophages. Heme caused macrophages to express the M1	Not measured

					phenotype, regardless of previous cell differentiation. Heme+Hx treatment prevented macrophage switching to M1 phenotype.	
Lin et al., Synergistic inflammation is induced by blood degradation products with microbial Toll-like receptor agonists and is blocked by hemopexin, (13)	Hemolytic disease (eg. SCD & ICH), 3 µmol/L hemin or 300µg/mL Hb treatment	n/d<br>C57BL/6 (WT), C3H/HeN, C3H/HeJ, MyD88$^{-/-}$ mice; BMDMs only<br>n/d<br>n/d<br>24h	TLR1, TLR2, TLR3, TLR4, TLR7, TLR9	30–1000µg/mL Hb, 1–3 µmol/L hemin, LPS, TLR4 agonist), Pam3Cys (TLR1/TLR2 agonist), poly I:C (TLR3 agonist), loxoribine (TLR7 agonist), CpG DNA (TLR9 agonist), human and murine hemopexin (hHx and mHx)	Treatment of BMDMs with TLR agonists (LPS, Pam3Cys, Poly I:C, loxoribine, CpG)+Hb groups had higher inflammatory cytokines expression than respective TLR agonist-only groups. Macrophages produced greater amounts of inflammatory cytokines in response to E. coli+Hb and S. aureus+Hb than the respective bacteria alone, with a greater difference with greater amounts of bacteria or longer incubation periods. Expression of inflammatory cytokines in response to TLR agonists (except LPS) did not decrease in TLR4$^{-/-}$ mice compared to WT. LPS+hemin ($p<0.001$), Lox+hemin ($p<0.01$), Poly I:C+hemin ($p<0.01$) showed increased expression of TNF, even	n/d (In vitro only)

					in TLR$^{-/-}$ and MyD88$^{-/-}$ mice. LPS+hemin showed decreased TNF expression in MyD88$^{-/-}$ mice compared to WT. mHx and hHx attenuate increased TNF expression caused by LPS+Hb and E. coli+Hb.	
Belcher et al., Identification of a Heme Activation Site on the MD-2/TLR4 Complex, (14)	Hemolytic disease (eg. SCD & ICH), incubation with 10μM heme	n/d<br>CHO cells, HEK293 cells<br>n/d<br>100% F (CHO), n/d (HEK293)<br>6h	TLR4	Control (1% FBS), heme, LPS (10ng/ml), Heme+LPS	MD-2 binds to heme, as found through pulldown assay. Results were confirmed by a peak at ~414 nm found in absorbance spectroscopy. Heme and LPS increased NFκB luciferase reporter response compared to control ($p<0.01$) but not if CD12, MD-2, or TLR4 was absent. NFκB reporter response to heme increased in Y36A mutant MD-2 ($p<0.01$) compared to WT. Reporter response decreased in W23A ($p<0.01$) and Y34A ($p<0.01$) mutant MD-2.	n/d (in vitro only)
Yoshizaki et al., Tranexamic acid reduces heme cytotoxicity via the TLR4/TNF axis and ameliorates	SCI, contusion injury at T9 vertebrae using impactor machine or heme/autologous blood injections	8–10wk<br>C57BL/6 N mice<br>92 mice<br>100% F<br>1wk	TLR4	SCI, sham (laminectomy only), TXA (1mg/g), heparin (1U/g), vehicle (saline), 125 or 250μM heme, autologous packed or lysed RBCs (2μl)	SCI+TXA group showed smaller intralesional RBC volume and bleeding area ($p<0.05$ each), as well as greater LFB staining ($p<0.05$) vs. the SCI+vehicle group. SCI+heparin group	SCI+TXA group had better functional outcomes than the SCI+vehicle group as

functional recovery after spinal cord injury, (15)	at the same location				showed larger intralesional RBC and bleeding area (p<0.05 each) and less LFB staining vs. SCI+vehicle group. Lysed RBCs had more heme content (p<0.05) and resulted in less LFB staining than packed RBCs. SCI group had a higher expression of Hpx and HO1 than the naive group. TLR4 expression was increased in microglia in Heme (p<0.05) and Lysed RBC (p<0.05) groups compared to the saline group. TLR4 expression scaled with increasing heme concentration. Expression of TLR4 was primarily in the area around the lesion and in microglia. SCI+TXA group contained fewer apoptotic cells (p<0.05), whereas the SCI+Heparin group had more (p<0.05) than SCI+vehicle. SCI+TXA group demonstrated less expression of the TLR4/MyD88 pathway than the SCI+vehicle, whereas SCI+heparin	measured through open field score, grip test, stride/swim tests (p<0.05 for each). Conversely, SCI+heparin had worse functional outcomes.

					demonstrated increased expression.	
Sudan et al., TLR4 activation alters labile heme levels to regulate BACH1 and heme oxygenase-1 expression in macrophages, (16)	Hemolytic disease (eg. SCD & ICH), incubation with (heme 10μM)	n/d<br>C57BL/6J (WT) mice, BACH1$^{-/-}$, and Nrf2$^{-/-}$ mice, hMDM<br>n/d<br>100% M (mice only)<br>48h	TLR4	LPS (TLR4 agonist; 1μg/ml), LTA (TLR2 agonist; 5μg/ml), heme (10μM), SA (heme inhibitor; 1mM), TAK-242 (TLR4 inhibitor; 10μg/ml), CORM-401 (carbon monoxide releaser; 50μM), and iCORM-401 (negative control; 50μM)	LPS treatment of murine BMDM resulted in a higher expression of HO1 ($p<0.01$), Nrf2 ($p<0.05$), and NQO1 ($p<0.05$) and lower expression of BACH1 ($p<0.001$) compared to control after 3h incubation. Heme also demonstrated higher expression of HO1 ($p<0.01$), Nrf2 ($p<0.05$), and NQO1 ($p<0.05$), as well as lower expression of BACH1 ($p<0.001$) than control. LPS stimulation resulted in higher labile heme levels ($p<0.05$) than control.<br>Nrf2$^{-/-}$ groups had a lower expression of HO1 and labile heme than WT groups. LPS and heme treatment still resulted in increased HO1 and labile heme expression. Nrf2$^{-/-}$ groups had higher BACH1 expression than WT. BACH1$^{-/-}$ control groups had higher HO1 and Nrf2 expression than WT control and neither LPS nor heme caused changes in expression.	n/d

			LPS treatment of hMDM resulted in downregulation of HO1 ($p<0.001$), upregulation of BACH1 ($p<0.001$), upregulation of Nrf2 ($p<0.001$), and downregulation of labile heme ($p<0.001$). The effect reached a peak at 4h incubation. Heme treatment of hMDM resulted in upregulation of HO1 ($p<0.001$), downregulation of BACH1 ($p<0.001$), and upregulation of Nrf2 ($p<0.001$). Treatment of hMDMs with TAK-242 resulted in increased labile heme expression ($p<0.001$), decreased TNF-α expression ($p<0.001$), decreased BACH1 expression ($p<0.001$), and increased HO1 expression ($p<0.01$) compared to LPS alone. No difference in expression in heme treatment. No difference in Nrf2 expression in any groups. CORM-401 treatment of hMDMs resulted in upregulation of Nrf2 ($p<0.001$), downregulation of	

					BACH1 ($p<0.001$), and upregulation of HO1 ($p<0.001$). Effects overrode the effects of LPS because labile heme expression was increased in the CORM-401+LPS group compared to control+LPS group ($p<0.001$) and compared to the no treatment group ($p<0.001$). hMDM groups treated with SA had a lower expression of HO1 ($p<0.001$) and upregulation of BACH1 ($p<0.05$) compared to the control group.	
Chen et al., RNF216 mediates neuronal injury following experimental subarachnoid hemorrhage through the Arc/Arg3.1-AMPAR pathway, (17)	SAH, 0.35mL autologous blood to prechiasmatic cistern	16–18 embryonic days (in vitro), Not specified (in vivo)<br>Sprague–Dawley rats<br>n=30 (in vivo)<br>n/d<br>24h (in vivo and in vitro)	All (RNF21 6 ubiquiti nates and degrade s all TLRs)	In vivo: sham, SAH (0.35mL autologous blood), siRNF216 (5μl), siARC (5μl), AMPA; In vitro: control, OxyHb, siRNF216, siARC, NBOX, NASPM, AMPA	In vitro: neurons treated with OxyHb had greater expression of RNF216 at 3h, 6h, and 24h ($p<0.05$ for each) than control but not 12h or 48h. The OxyHb+siRNF216 group had decreased neuron apoptosis ($p<0.05$) and neuronal toxicity ($p<0.05$) compared to the OxyHb group. The OxyHb group had increased expression of GluR1 ($p<0.05$) and GluR2 ($p<0.05$). Increased expression was attenuated in OxyHb+siRNF216.	The SAH group had higher neurological scores than the SAH+siRNF21 6 group ($p<0.05$).

OxyHb+NBQX and OxyHb+NASPM groups had decreased apoptosis and neuronal toxicity compared to the OxyHb group ($p<0.05$ for each). Decrease in apoptosis was partially reversed in the OxyHb+siRNF216+AMPA group ($p<0.05$). OxyHb group had increased expression of Arc from 3h to 6h ($p<0.05$ for each), but not 12h and later. The OxyHb+siRNF216+siArc group had increased neuronal toxicity ($p<0.05$) and apoptosis ($p<0.05$) compared to the OxyHb+siRNF216 group. The OxyHb+siArc group had increased GluR1 ($p<0.05$) and GluR2 ($p<0.05$) expression. The OxyHb group had an increase of intracellular Ca^{2+} in neurons. The increase was attenuated by NASPM and siRNF216. In vivo: SAH group had increased RNF216 after 6h compared to sham, reaching a peak at 12h ($p<0.05$).

Yang et al., Luteolin alleviates neuroinflammation via downregulating the TLR4/TRAF6/NFκB pathway after intracerebral hemorrhage, (18)	ICH, 100μl autologous blood to right basal ganglia	Embryonic day 18 (in vitro)<br>250−300g(in vivo)<br>Sprague−Dawley rats (in vivo), Sprague−Dawley rats microglial and neuron culture (in vitro)<br>n=96 (experiment 1), n=36 (experiment 2)<br>100% M<br>24h (in vivo and in vitro)	TLR4	Experiment 1 and 2: sham, ICH, ICH+vehicle, and ICH+luteolin; Experiment 3 (in vitro): luteolin (0, 5, 10, 25, 50, and 100μM) and OxyHb (10μM)	In vivo: ICH+20mg/kg luteolin group had less microglial activation ($p<0.01$), lower expression of inflammatory cytokines ($p<0.05$), and lower neuronal degradation ($p<0.01$) than ICH+vehicle. The SAH+10mg/kg group increased ubiquitination (not quantified) and decreased degradation ($p<0.05$ after 61 centigrade) of TRAF6. In vitro: OxyHb+10mg/kg luteolin and OxyHb+20mg/kg luteolin had lower inflammatory cytokine expression and more viable microglia than the OxyHb+DMSO group. OxyHb+10mg/kg luteolin and OxyHb+20mg/kg luteolin had lower expression of the TLR4/TRAF6 pathway than OxyHb+DMSO. Treatment with 10μM luteolin prevented p65 translocation in BV2 cells treated with 10μM OxyHb.	ICH+20mg/kg luteolin had longer rotarod performance at 24h, 36h, and 48h ($p<0.01$ for each) and faster adhesive test at 24h, 36h, and 48h ($p<0.01$) than ICH+vehicle. A similar smaller difference in the ICH+10mg/kg group, but not in the ICH+5mg/kg group.
Zhong et al., Interleukin-23	ICH	20−24g<br>C57BL/6 (WT), IL-17$^{-/-}$,	TLR2/TLR4	In vivo: sham-operated, ICH (20μl autologous	In vivo: Macrophages and CD3, CD4, CD45,	ICH+IL-23p19$^{-/-}$ had a

Secreted by Activated Macrophages Drives γδT Cell Production of Interleukin-17 to Aggravate Secondary Injury After Intracerebral Hemorrhage, (19)	IL-23p19$^{-/-}$, T−cell receptor γδ$^{-/-}$ (γδT$^{-/-}$), TRIF $^{-/-}$, TLR2$^{-/-}$, TLR4$^{-/-}$, MyD88 $^{-/-}$, and Rag1$^{-/-}$ mice (in vivo); HEK−293 cells, BMDCs (in vitro) 100% M 7d	blood), vehicle (DMSO or liposomes), CLPs (0.2mL/20–25 g), fingolimod (1mg/kg, single or repeated administration), sparstolonin B (SsnB, 5mg/kg); In vitro: Hb, hemin, bilirubin, $Fe2+$, $Fe3+$ (all at 5μM), SsnB (1, 10, or 50μM)	and γδT positive cells infiltrate into the brain after ICH. Macrophage infiltration peaks at 1d post-ICH, whereas T-lymphocyte infiltration peaks at 4d. TLR2/TLR4 heterodimers form on infiltrating macrophages. ICH+IL-23p19$^{-/-}$ had less BWC and less neuron apoptosis than ICH+WT at 1d, 4d, and 7d ($p<0.05$ for each). IL-23p19 is only expressed in detectable amounts in macrophages. Expression peaks at 1d post-ICH. ICH+CLP group had lower macrophage infiltration ($p<0.01$) but no significant difference in lymphocyte infiltration compared to ICH+WT. ICH+CLP group had less BWC than ICH+WT at 1d, 4d, and 7d. ICH+Fingolimod group had less lymphocyte infiltration but no s.d. in macrophage infiltration compared to ICH+vehicle. The ICH+Rag$^{-/-}$ group had lower BWC than ICH+WT, but the	lower NDS than ICH+WT at 1d, 4d, and 7d ($p<0.05$ for each). NDS was lower in the ICH+CLP group than the ICH+vehicle group at 1d, 4d, and 7d. ICH+fingolimo d group had lower NDS scores than ICH+vehicle ($p<0.05$). The ICH+Rag$^{-/-}$ group had a lower NDS than ICH+WT, but the difference disappeared when treated with WT CD3 lymphocytes. Chimeric WT+TLR2$^{-/-}$/T LR4$^{-/-}$ bone marrow mice had lower NDS than WT, but the difference disappeared in TLR2$^{-/-}$/TLR4$^{-/-}$

difference disappeared when treated with WT CD3 lymphocytes. ICH+fingolimod group had lower BWC than ICH+vehicle ($p<0.05$). Chimeric WT+TLR2$^{-/-}$/TLR4$^{-/-}$ bone marrow mice had lower BWC and fewer apoptotic cells than WT, but the difference disappeared in TLR2$^{-/-}$/TLR4$^{-/-}$+WT BM mice. In vitro: IL-23p19 expression was highest in BMDCs treated with 5μM Hb at 3 h. IL-23p19 expression in BMDCs was lowest in TLR2$^{-/-}$/TLR4$^{-/-}$ cells and MyD88$^{-/-}$ cells. TLR2$^{-/-}$ and TLR4$^{-/-}$ cells had a lower expression to a lesser extent, but TRIF$^{-/-}$ cells had no change in expression. ICH+γδT$^{-/-}$ group had lower BWC and less expression of inflammatory cytokines than ICH+WT. The ICH+SsnB group had decreased BWC ($p<0.05$) and lower expression of inflammatory cytokines such as IL-23p19.	+WT BM mice ($p<0.05$). The ICH+γδT$^{-/-}$ group had a lower NDS than ICH+WT ($p<0.05$). ICH+SsnB group had a lower NDS than ICH+vehicle ($p<0.05$).

Merle et al., P-selectin drives complement attack on endothelium during intravascular hemolysis in TLR-4/heme dependent manner, (20)	Hemolytic disease (eg. SCD & ICH), heme (40µmol/kg) or phenylhydrazine (PHZ, 900µmol/kg) injected intraperitoneally	8wks<br>C57BL/6 (WT), $C3^{-/-}$, and $TLR4^{-/-}$ mice (in vivo); HUVEC (in vitro)<br>n/d<br>Each group matched by sex<br>3d after first heme/PHZ administration	TLR4	In vivo: vehicle (PBS, 100µl, administered twice, 24h apart), heme (40µmol/kg, same administration), phenylhydrazine (PHZ, 900µmol/kg, same administration), human hemopexin (Hx, 40µmol/kg, administered 1h before heme or PHZ), irrelevant IgG antibodies (Irr lg, administered 5min before heme/PHZ), anti-C5 antibodies (α-C5, same administration), anti–P-selectin antibodies (α-P-sel, same administration) In vitro: heme (25, 100µM), LPS (25µM), TAK-242 (25µM)	In vivo: Heme+$TLR4^{-/-}$ ($p<0.01$) and PHZ+$TLR4^{-/-}$ ($p<0.05$) groups had lower C3 activation than the respective WT groups. The PHZ+Hx group had reduced staining of C3 vascular deposits. Only PHZ resulted in increased expression of alanine aminotransferases (ALT, liver failure marker) ($p<0.0001$) than vehicle. The increase was attenuated in $TLR4^{-/-}$ mice ($p<0.05$) and $C3^{-/-}$ mice ($p<0.0001$). Expression of NGAL in response to heme and PHZ was attenuated in TLR4/- and C3/- mice. α-C5 antibodies attenuated increased C5a expression caused by PHZ compared to irrelevant antibodies ($p<0.0001$). Heme+α-P-sel antibody and PHZ+α-P-sel antibody groups had reduced expression of NGAL and activation of C3 compared to heme and PHZ groups, respectively. In vitro: heme activated the TLR4	n/d

					pathway in HUVEC. Activation of the TLR4 pathway by heme resulted in complement protein (C3, C5) deposits in HUVEC. Heme, but not LPS, resulted in C3 and C5 activation after TLR4 inhibition. C3 binds P-selectin. C3 activation in response to heme was attenuated in an acidic (pH 2.5) environment ($p<0.01$). Attenuation disappeared after cotreatment with TAK-242. Treatment with α-P-sel antibodies did not result in attenuation by TAK-242 compared to irrelevant antibodies ($p<0.01$)	
Philip et al., TLR stimulation dynamically regulates heme and iron export gene expression in macrophages, (21)	Hemolytic disease (eg. SCD & ICH), incubation with hemin (0-1000µM) for 48h	6–8wk J774A.1 cell line, C75BL/6 (WT), $TLR4^{-/-}$, and $Ly96^{-/-}$ mice, BMDMs n/d 100% M 48h	TLR1, TLR2, TLR3	Hemin (0–1000µM, incubated for 24h), LPS (TLR4 agonist, 0–1000ng/mL incubated for 48h), Pam3Csk4 (Pam3, TLR1/2 agonist), polyinosinic:polycytidylic acid (pIC, TLR3 agonist)	Hemin increased the expression of Hmox1, Fpn1, and Ftl1 but not Flvcr. LPS upregulated Hmox1 and downregulated Ftl1 and Flvcr, but had dose-dependent effects on Fpn1 (decrease at small doses, increase at large doses) in J774 cells. Compared to M0 macrophages, M1 macrophages had a lower expression of Flvcr	n/d

3h-24h and Hmox1 at 6h, while having a higher expression of Hmox1 at 12 to 48h ($p<0.05$ for each). Compared to M0 macrophages, M2 macrophages only had a lower expression of Flvcr at 12h ($p<0.05$). Fpn1 was downregulated in both M1 and M2 phenotypes ($p<0.05$ for each). A decrease in Flvcr expression in BMDM treated with LPS and IFN-γ was attenuated in $TLR4^{-/-}$;$Ly96^{-/-}$ cells ($p<0.01$ for both) compared to WT. Upregulation of Hmox1 in cells treated with LPS was attenuated in $TLR4^{-/-}$;$Ly96^{-/-}$ ($p<0.001$) compared to WT; however, upregulation by Pam3 was not. Decrease in Fpn1 expression in BMDM treated with LPS and IFN-γ was attenuated in $TLR4^{-/-}$;$Ly96^{-/-}$ cells ($p<0.001$ for LPS, $p<0.01$ for IFN-γ) compared to WT. LPS activated transcription factors bind to the Flvcr promoter to increase expression.

Wang et al., miR-140-5p Attenuates Neuroinflammation and Brain Injury in Rats Following Intracerebral Hemorrhage by Targeting TLR4, (22)	ICH, Bacterial collagenase type IV (0.23U) to the right striatum	12 wk<br>200–220g<br>Sprague–Dawley rats (in vivo), PC12 cells (in vitro)<br>100% M<br>n=45	TLR4	Sham (1μL saline), negative control mimics (NC, 5μL), miR-140-5p mimics (20nmol/L to the right lateral ventricle, 3 days pre-ICH)	Endogenous miR-140-5p reached the lowest level of expression at 2d post-ICH ($p<0.01$ vs. sham); TLR4 reached the highest level of expression at 2d post-ICH ($p<0.01$ vs. sham). TLR4 was strongly inversely correlated with miR-140-5p ($p<0.01$). The effect of miR-140-5p on TLR4 expression was reversed in the mutant form of TLR4. miR-140-5p inhibitor resulted in increased expression of TLR4 ($p<0.01$). ICH+miR-140-5 had fewer apoptotic neurons than ICH+NC ($p<0.01$). ICH+miR-140-5 had a lower expression of TNF-α, IL-1β, and IL-6 (downstream products of TLR4) at 12h and 1d than ICH+NC ($p<0.01$ for each).	ICH+miR-140-5 had a lower NDS than ICH+NC ($p<0.01$).
Liu et al., MiR-146a Ameliorates Hemoglobin-Induced Microglial Inflammatory Response via TLR4/IRAK1/T	SAH, monofilament puncture of the bifurcation of the anterior and middle cerebral arteries	280–320g<br>Sprague–Dawley rats<br>n=32<br>100% M<br>24h (in vitro), 7d (in vivo)	TLR4	miR-146a transfection, miR-146a mimic (negative control) transfection	In vivo: Significant decrease in endogenous miR-146a expression ($p<0.001$) in the SAH group. In addition, a decrease in neuronal damage was found in rats treated with exogenous miR-146a	n/d

RAF6 Associated Pathways, (23)					(p<0.01). In vitro: miR-146a resulted in a slight decrease of proinflammatory markers early on after treatment and a slight increase of anti-inflammatory markers later (p<0.05). miR-146a also decreased expressions of TRAF6 and IRAK1, proteins within the TLR4-mediated pathway, at 1h and 4h, but not of TLR4 itself.	
Gao et al., Curcumin Mitigates Neuro-Inflammation by Modulating Microglia Polarization Through Inhibiting TLR4 Axis Signaling Pathway Following Experimental Subarachnoid Hemorrhage, (24)	SAH, 50µl heterologous blood was injected into the prechiasmatic cistern	25-28g C57BL/6J (WT) and $TLR4^{-/-}$ mice n=189 100% M 24h	TLR4	Curcumin (Cur, 50–200mg/kg)	The SAH+Cur group had less BWC (p<0.05) and apoptotic neurons (p<0.05) than SAH+Vehicle. The 100mg/kg dose showed the greatest reduction in TLR4 expression (p<0.01 vs. SAH+Veh). SAH+Cur group had less M1 phenotype (p<0.05) and more M2 (p<0.05) than SAH+Vehicle. SAH+Cur group had decreased expression of TLR4 and MyD88 and increased expression of IκB-α (p<0.05 vs. SAH+Veh for each). The SAH+Cur group had reduced expression of IL-1β, IL-6,	The SAH+Cur group had a lower NDS than SAH+Vehicle (p<0.001). The SAH+$TLR4^{-/-}$ group had a lower NDS than SAH+WT (p<0.001). The SAH+Cur+TLR4$^{-/-}$ mice had no change in TLR4, cytokine, M1/M2 expression, or BWC

					TNF-α, and iNOS (proinflammatory cytokines) and lower expression of TGF-β and IL-10 (anti-inflammatory cytokines) ($p<0.05$ vs. SAH+Veh for each). The SAH+TLR4$^{-/-}$ group had less TLR4 expression than SAH+WT ($p<0.001$). SAH+TLR4$^{-/-}$ group had fewer apoptotic neurons ($p<0.05$) and less BWC ($p<0.05$) than SAH+WT. SAH+Cur+TLR4$^{-/-}$ mice had no change in TLR4, cytokine, M1/M2 expression, or BWC compared to SAH+TLR4$^{-/-}$.	compared to SAH+TLR4$^{-/-}$.
Lisk et al., Hemoglobin-induced endothelial cell permeability is controlled, in part, via a myeloid differentiation primary response gene-88-dependent signaling mechanism, (25)	Hemolytic disease (eg. SCD & ICH)	n/d<br>HMEC-1<br>n/d<br>n/d<br>24h	TLR4	Oxyhemoglobin (HbFe2+, 500nM to 500μM), Methemoglobin (HbFe3+), Ferryl hemoglobin (HbFe4+), LPS (150 U)MyD88-siRNA, NFκB-siRNA, HIF-1α-siRNA, TAK-242 (TLR4 inhibitor), LPS (150U), CuZn SOD, 150U, catalase (150U), dimethyloxalylglycine (DMOG, 30μg)	All forms of Hb induced expression of NFκB and HIF, with HbFe4+ inducing the highest level of expression. The most effective concentration was between 100 and 300μM for each Hb. Effects of every type of Hb on HIF-1α and HIF-2α mRNA expression was reversed by NFκB-siRNA. MyD88-siRNA treatment with TAK-242 had no significant impact on the change in NFκB and HIF	n/d

					activity induced by each type of Hb. Cotreatment with SOD and catalase attenuated the change in NFκB and HIF activity induced by Hb. There was no significant difference in oxidative stress induced by each type of Hb. Cotreatment with SOD and catalase attenuated the change in NFκB and HIF activity induced by Hb. There was no significant difference in oxidative stress induced by each type of Hb.	
Lee et al., Response of neutrophils to extracellular haemoglobin and LTA in human blood system, (26)	Hemolytic disease (eg. SCD & ICH), incubation with metHb (0.5mg/mL)	n/d<br>n/d<br>HEK293 expressing human TLR2/TLR9, Human monocytes, leukocytes, neutrophils<br>n/d<br>n/d<br>6h	TLR2/TLR9	metHb (0.5mg/ml), LTA (100ng/ml), metHb+LTA	NFκB activation increased by metHb and LTA, but only in hTLR2 expressing cells ($p<0.05$ each). LTA+ 0.5mg/mL MetHb has more NFκB activation than LTA alone ($p<0.05$). NFκB is activated by ODN, but not metHb, in hTLR9 expressing cells. metHb increased ROS production in neutrophils ($p<0.05$) and monocytes ($p<0.05$) but not lymphocytes compared to control. metHb+LTA had increased ROS	n/d

production in neutrophils but not monocytes compared to metHb ($p<0.05$). IL-8 expression was increased by metHb and LTA in PBMC and WBC compared to control, but metHb+LTA had no synergistic effect. metHb and LTA both result in an increase in ROS, IL-8, and TNF-α in neutrophils, and display a synergistic effect in metHb+LTA group ($p<0.05$ vs. metHb alone). Cotreatment with TLR2 antibodies resulted in a reversal of metHb and LTA-induced increase. TNF-α expression is positively correlated with metHb concentration ($p<0.05$)

Table S2. Clinical Table

Reference	Disease Studied & Model	Avg. Age, Population Size, Sex, Location	Study Design & Duration	Exclusion Criteria	Treatment/ Dosage/ Delivery	Anatomical/ Biochemical Outcomes	Functional/ Behavioral Outcomes
Sokół et al., Soluble Toll-Like Receptors 2 and 4 in Cerebrospinal Fluid of Patients with Acute Hydrocephalus following Aneurysmal Subarachnoid Haemorrhage, (27)	SAH	56.78±17.7y N=18 (+8 controls) [Small sample size] 72.2% M Heliodor Święcicki Clinical Hospital, Poznan University of Medical Sciences (Poznan, Poland)	Prospective case-control study 12/2013 – 6/2015	<18y, pregnancy, history of CNS disease (meningitis, stroke), active infection of CNS, systemic disease (diabetes mellitus, rheumatoid arthritis, malignancy, cirrhosis, renal failure), and patients who underwent clipping.	3 sets of lumbar punctures to draw CSF	There were no sTLRs detected in the controls. The level of sTLR4 in the CSF increased over time post-SAH ($p<0.01$). sTLR2 levels were highest in the CSF 5d post-SAH ($p<0.01$ compared to days 0–3). The CSF Hb levels correlated with 5d TLR2 ($p<0.05$) and 5d TLR4 ($p<0.05$) levels.	GOS (patient outcome scale) was not correlated with any variable except WFNS scale, HH scale, and GCS scale ($p<0.001$ for each).
Li et al., Serum levels of matrix metalloproteinase 9 and toll-like receptor 4 in acute aortic dissection: a case-control study, (28)	Aortic dissection	56.7±12.4y 58.6±12.1y (controls) N=88 (+88 matched controls) 72.7% M & 73.9% M (controls); First Hospital of China Medical University (Shenyang, China) [Small sample area]	Prospective case-control study 3/2017 – 1/2018	Chronic aortic dissection, coronary heart disease, congenital heart disease, severe vascular stenosis, autoimmune diseases, severe organ failure, infectious diseases, malignant tumors, hematological disease, previous aortic surgery, and use of NSAIDs or steroids.	Blood serum analysis	Both AAD types had higher levels of creatinine and homocysteine, higher WBC count, and lower levels of Hb than controls ($p<0.05$ for each). The type A group had higher creatinine and D-dimer levels than the type B group ($p<0.05$ for both). Both AAD groups had significantly higher serum MMP9 and	Using MMP9 and TLR4 in a diagnostic test for AAD had an AUC of 0.837 (sensitivity=60.2%, specificity=94.3%). Change in MMP9 and TLR4 over time were not measured RP and D-dimer levels were not measured in the control group.

						TLR4 levels (p<0.05 for each). There was no s.d. between type A and type B. MMP9 (OR = 1.010 per unit increase, 95% CI=1.006–1.013, p<0.001) and TLR4 (OR=1.393 per unit increase, 95% CI= 1.232–1.576, p<0.001) were both associated with increased risk for AAD. Significant associations between MMP and TLR4 (r=0.518, p<0.001); CRP and MMP9 (r=0.237, p=0.019); CRP and TLR4 (r=0.436, p<0.001). No significant association between MMP9 and TLR4.	
Ma et al., Toll-like receptor 4 (TLR4) is correlated with delayed cerebral ischemia (DCI) and poor prognosis in aneurysmal subarachnoid hemorrhage, (29)	Aneurysmal SAH	54.3±3.3y N=30 (+20 matched controls) [Convenience Sample] 63.3% M & 68.8%M (controls) Department of Neurosurgery of Henan	Prospective case-control study 10/2013 – 10/2014	Nonaneurysmal SAH, chronic infection, hydrocephalus, interventional treatment or operation before admission, previous head trauma, neurological disease including ischemic or hemorrhage stroke, use of antiplatelet or anticoagulant	Blood serum analysis	The PBMC of TLR4, WBC count, serum IL-6, and serum TNF-α were all elevated after aSAH, reaching peak at 1d post-aSAH and decreasing thereafter. Patients with DCI had a higher TLR4 expression than	Patients with high Hunt-Hess grades (IV-V) had higher TLR4 expression than patients with lower Hunt-Hess grades (I-III) (p<0.05). Patients with high modified Rankin score (0–3) had higher TLR4 expression than patients with lower scores (4-5) (p<0.01). PBMC TLR4 expression

	Provincial People's Hospital (Zhengzhou, China)	medication, and any other prior systemic disease (e.g., uremia, liver cirrhosis, malignancy, chronic heart or lung disease, diabetes mellitus and hypertension).	patients without DCI ($p<0.01$). TLR4 expression was positively correlated with IL-6 expression ($r=0.876$, $p<0.001$) and TNF-α ($r=0.656$, $p<0.001$) in patients.	had 86.4% sensitivity, 70.6% specificity for predicting DCI, and 68.8% sensitivity, 78.6% specificity for predicting poor neurological outcome at 3 mo.

Abbreviations: AAD = acute aortic dissection; AUC = area under the curve; aSAH = aneurysmal subarachnoid hemorrhage; ATX = astaxanthin; BBB = blood brain barrier; BMDC = bone marrow-derived dendritic cells; BMDM = bone marrow-derived macrophages; BWC = brain water content; CHO = Chinese hamster ovary; CI = confidence interval; CNS = central nervous system; CORM = carbon monoxide-releasing molecule; CLPs = clodronate liposomes; CSF = cerebrospinal fluid; DCI = delayed cerebral ischemia; D/D = natural point mutation; GCS = Glasgow coma scale; GOS = Glasgow outcome scale; Hb = hemoglobin; HH = Hunt & Hess scale; HIF = hypoxia inducible factor; hMDM = human monocyte-derived macrophages; HO1= heme oxygenase 1; HUVEC = human umbilical vein endothelial cells; ICH = intracerebral hemorrhage; IL = interleukin; LFB = Luxol fast blue; LPS = lipopolysaccharides; NDS = neurological deficit score; NSAID = nonsteroidal anti-inflammatory drug; OR = odds ratio; PBMC = LTA = lipoteichoic acid; peripheral blood mononuclear cell; PBS = phosphate buffered saline; pMCAO = permanent middle cerebral artery occlusion; POD = postoperative day; RBC = red blood cell; ROS = reactive oxygen species; SA = succinylacetone; SAH = subarachnoid hemorrhage; SCI = spinal cord injury; SCD = sickle cell disease; SOD = superoxide dismutase; sTLR = soluble toll-like receptor; TLR = toll-like receptor; TNF = tumor necrosis factor; TXA = tranexamic acid; WBC = white blood cell; WFNS = World Federation of Neurological Surgeons; WT = wild type; YFP = Yellow Fluorescent Protein
